# Cell Counts, rather than Proportion, of CD8/PD-1 Tumor-Infiltrating Lymphocytes in a Tumor Microenvironment Associated with Pathological Characteristics of Chinese Invasive Ductal Breast Cancer

**DOI:** 10.1155/2019/8505021

**Published:** 2019-03-31

**Authors:** Qingkun Song, Feng Shi, Maya Adair, Hong Chang, Xiudong Guan, Yanjie Zhao, Yuchen Li, Guangjiang Wu, Jiangping Wu

**Affiliations:** ^1^Department of Science and Technology, Beijing Shijitan Hospital, Capital Medical University, Beijing 100038, China; ^2^Beijing Key Laboratory of Cancer Therapeutic Vaccines, Beijing 100038, China; ^3^Department of Pathology, Beijing Shijitan Hospital, Capital Medical University, Beijing 100038, China; ^4^University of the Incarnate Word, San Antonio, TX 78209, USA; ^5^Beijing Tiantan Hospital, Capital Medical University, Beijing 100050, China; ^6^Department of Medical Oncology, Beijing Shijitan Hospital, Capital Medical University, Beijing 100038, China; ^7^Sid Faithfull Brain Cancer Research Laboratory, QIMR Berghofer Medical Research Institute, Locked Bag 2000 Royal Brisbane Hospital, QLD 4029, Australia; ^8^Department of Infection Control, Beijing Shijitan Hospital, Capital Medical University, Beijing 100038, China; ^9^Department of Cancer Research, Beijing Shijitan Hospital, Capital Medical University, Beijing 100038, China

## Abstract

**Objective:**

This study is aimed at investigating the association of exhausted CD8^+^ tumor-infiltrating lymphocytes with clinic-pathological factors.

**Methods:**

133 patients diagnosed with primary invasive ductal breast cancer were recruited into the cross-sectional study consecutively. Immunohistochemistry was used to detect biomarker expression on formalin-fixed and paraffin-embedded sections. Double staining of CD8 and PD-1 was conducted on lymphocytes.

**Results:**

The proportion of CD8^+^/PD-1^−^ TILs was 16% among patients with axillary lymph node metastasis, significantly lower than those without metastasis (24%). The expression of CK7, CK20, or Ki-67 was not related with the proportion of phenotypes of CD8/PD-1 TILs. Younger patients had more cell counts of CD8^+^/PD-1^−^ TILs than elderly patients (18/HPF vs. 9/HPF, *p* < 0.05). Patients with axillary lymph node metastasis had less CD8^+^/PD-1^−^ TILs than those without metastasis (11/HPF vs. 27/HPF, *p* < 0.05). Median counts of CD8^+^/PD-1^−^ TILs among patients with CK20 and E-Cad expression were 33/HPF and 14/HPF, significantly higher than those among patients with negative CK20 (16/HPF) and E-Cad expression (6/HPF). Ki-67 index had a significant correlation with cell counts of CD8^+^/PD-1^+^ TILs and CD8^+^/PD-1^−^ TILs, and the correlation coefficients were 0.19 and 0.21 (*p* < 0.05), respectively.

**Conclusion:**

The proportion of CD8^+^/PD-1^−^ TILs was related with metastatic status of the axillary lymph node but cell counts of CD8^+^/PD-1^−^ TILs were related with metastatic status of the axillary lymph node and expression of CK7, CK20, E-Cad, and Ki-67. Absolute cell counts, not proportion of CD8/PD-1 TILs, were more likely to distinguish clinic and pathologic characteristics of breast cancer.

## 1. Introduction

Breast cancer (BC) is the most common female cancer in China, with the incidence and mortality rate increasing to 28.77/10^5^ (279,000 new cases) and 6.35/10^5^ (66,000 new cases) in 2014, respectively [1]. Though the incidence rate in China was lower than that in the United States, Chinese patients have a lower hormone receptor and higher human epidermal growth factor receptor 2 (HER2) than Western women [2]. BC in Chinese women is more aggressive, and a clinical study is significant in Chinese BC patients.

Tumor-infiltrating lymphocytes (TILs) improved the clinical efficacy of chemotherapy [3] and showed a correlation with a better prognosis of BC [4, 5]. CD8^+^ T lymphocytes are effector T cells that present a cytotoxic function and tie to a longer survival among BC patients [5]. However, programmed cell death protein 1 (PD-1) is an inhibitory molecular T cell and suppresses immune functions of effector T cells [6]. The interaction between PD-1 and programmed cell death ligand 1 receptor (PD-L1) activated the inhibition of immunity [7], and the T cells with a positive PD-1 expression were termed as “exhausted T cells” [8]. A poorer prognosis of BC was reported to be related with a high level of PD-1^+^ TILs [9]. Compared with normal tissues, malignant tissues had a higher PD-1 expression on T cells and PD-1^+^/CD8^+^ TILs displayed an impaired antitumor immunity that produces less IL-2 and IFN-*γ* and compromises the control of tumor growth [10, 11]. This study is aimed at investigating the status of exhausted and unexhausted CD8^+^ effector TILs and the correlation with clinic-pathological characteristics among Chinese BC patients.

## 2. Methods

### 2.1. Ethical Approval and Informed Consent

All procedures performed in this study involving human participants were approved by the ethical committee of Beijing Shijitan Hospital, Capital Medical University, in accordance with the ethical standards of the 1964 Helsinki declaration and its later amendments.

As a retrospective study, the informed consent was waived.

### 2.2. Patients

133 patients diagnosed with invasive ductal BC were recruited into this cross-sectional study. Patients received surgeries at the Department of Breast Surgery, Beijing Shijitan Hospital, Capital Medical University, from January 1, 2012, to December 31, 2013, consecutively. All of the cases were pathologically confirmed with primary invasive BC at an operable stage.

### 2.3. Tissue Collection

The surgical specimen was prepared after operation, fixed in 4% neutral formaldehyde, and embedded in paraffin (FFPE), and the staining of hematoxylin and eosin was processed. The histopathological features were determined on a series of 4 *μ*m thick sections from each specimen, and Nottingham modification of the Bloom–Richardson system was used to classify the histological grade of BC at diagnosis.

### 2.4. Immunohistochemistry (IHC)

IHC was used to detect the biomarkers on FFPE sections, and the detailed procedures were described previously [12]. Monoclonal antibodies against PD-1 (mouse anti-human, # UMAB199), CD8 (rabbit anti-human, # SP16), CK7 (rabbit anti-human, #EP16), CK20 (rabbit anti-human, #EP23), Ki-67 (mouse anti-human, #MIB1), and E-Cadherin (E-Cad) (mouse anti-human, #NCH-38) were purchased from Beijing Zhongshan Golden Bridge Biotechnology Co. Ltd. Sections were baked for dehydration at 60°C in an oven for 60 min, dewaxed for 20 min, and washed in 100%, 100%, 95%, and 75% alcohol for 2 min, respectively, then washed with PBS by 5 times, 2 min each time. Antigen retrieval was carried out using the EnVision™ FLEX Target Retrieval Solution for 2 min and 30 sec; cooled to room temperature for 20 min; washed with PBS by 5 times, 2 min each time; and then incubated with 3% H_2_O_2_ at room temperature for 15 min, washed with PBS by 5 times, 2 min each time; sealed with 5% serum at 37°C for 15 min; discarded with a moderate primary antibody added at 4°C for a night; washed with PBS by 5 times, 2 min each time; and added with DAB for 5-10 min (PD-1, CK7, CK20, Ki-67, and E-Cad) and AP-red for 10-15 min (CD8). The counterstain was conducted on slides with hematoxylin.

### 2.5. IHC Scoring

Two pathologists evaluated the average TILs within the borders of the invasive tumor. TILs in areas of crush artifacts, necrosis, regressive hyalinization, and core biopsy were excluded. The pathologists evaluated mononuclear cells including lymphocytes and plasma cells, but not the polymorphonuclear leukocytes. 10 high-power fields (HPF, ×400) were randomly selected on IHC sections to count the average number of TILs.

A positive expression of CK7 and CK20 was defined as a clear brown cytoplasm of BC cells. Positive E-Cad expression was presented as a brown cytomembrane of BC cells. Ki-67 expression was defined as a brown nucleus in BC cells, and the Ki-67 index was measured as the proportion of Ki-67 expression among 1000 BC cells. Positive CD8 expression was classified as a red cytomembrane of lymphocytes. The proportion of CD8^+^ TILs was estimated among 1000 TILs. A positive PD-1 expression was recorded as a brown cytoplasm in lymphocytes. The expression rate of PD-1 was estimated with 1000 TILs. Double staining of CD8/PD-1 was presented as a red cytomembrane and brown cytoplasm of lymphocytes. The expression rate of CD8/PD-1 was estimated among 100 CD8^+^ TILs in both intratumoral and stromal locations.

## 3. Statistical Analysis

All analyses were conducted with SPSS software (version 17.0). The median and interquartile range (IQR) were used to describe TIL counts. Age was transformed in categorical scale by a median of 55. The difference in TIL phenotypes was estimated by Wilcoxon tests between age, nerve invasion, vascular invasion, and axillary lymph node metastasis groups. The association of TIL phenotypes with histological grade was estimated by Spearman correlation tests. Wilcoxon tests were used to estimate the difference of TIL phenotypes between positive and negative expressions of CK7, CK20, and E-Cad. The Spearman correlation test was used to measure the relationship between Ki-67 index and cell counts of TIL phenotypes. All analyses were two-sided, and the significance level was 0.05.

## 4. Results

The average age of included patients was 57.8 years (Table 1). 11.3% patients were diagnosed at histological grade I, 32.3% patients had vascular invasion, 17.3% patients had nerve invasion, and 54.2% patients had axillary lymph node metastasis (Table 1). The average Ki-67 index was 30% (Table 1). The expression rate of E-Cad, CK20, and CK7 was 94.9%, 8.2%, and 86.8%, respectively (Table 1).

**Figure 1:**
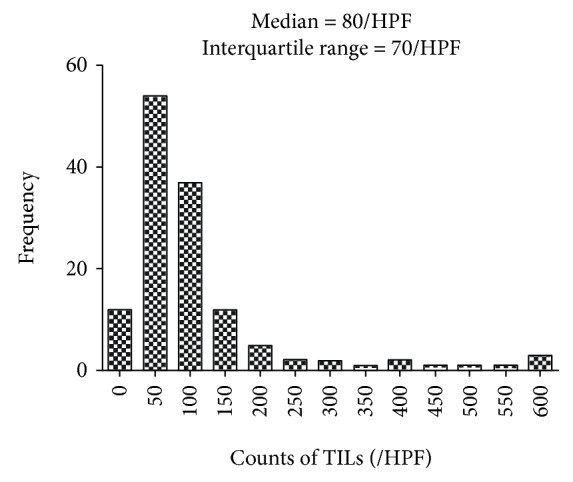
Distribution of TILs in the microenvironment of breast cancer.

Median cell counts of TILs were 80/HPF, with the IQR being 70/HPF (Figure 1). The proportion of CD8^+^ TILs had a significant association with axillary lymph node metastasis that the proportion of CD8^+^/PD-1^−^ TILs was 16% among patients with axillary lymph node metastasis, significantly lower than that among those without metastasis (24%, *p* < 0.05, Table 2). Age had a significant relationship with cell counts of CD8^+^ TILs: the median of CD8^+^ TILs was 24/HPF and 12/HPF in the groups of age equal to or younger than 55 and older than 55, respectively (*p* < 0.05, Table 3). However, the association was significant with cell counts of CD8^+^/PD1^−^ TILs, but not CD8^+^/PD1^+^ TILs (Table 3). The median count of CD8^+^/PD1^−^ TILs was 18/HPF among cases equal to or younger than 55 and 9/HPF among cases older than 55 (*p* < 0.05, Table 3). Occurrence of axillary lymph node metastasis was significantly related with cell counts of CD8^+^/PD1^−^ TILs, median cell counts being 27/HPF among cases with axillary lymph node metastasis, significantly higher than those among cases without axillary lymph node metastasis (11/HPF, Table 3). Histological grade, vascular invasion, and nerve invasion metastasis did not have any significant associations with cell counts of phenotypes of CD8/PD-1 TILs (Table 3).

Expressions of CK7, CK20, E-Cad, or Ki-67 were not associated with proportions of phenotypes of CD8/PD-1 TILs (Table 4).

**Figure 2:**
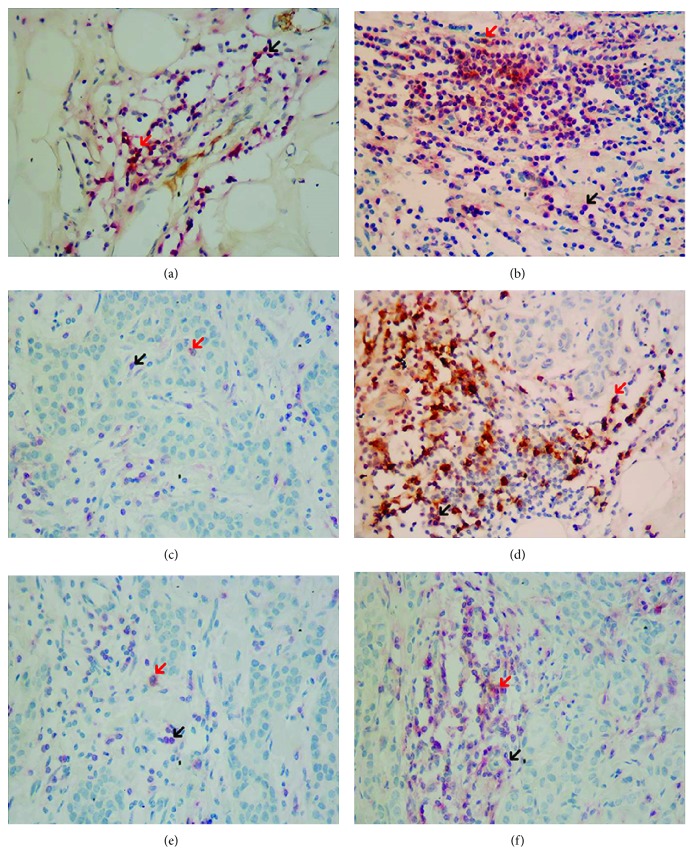
Expression of E-Cad, Ki-67, and CK-20 and counts of phenotypes of CD8/PD-1 TILs. (a) Patients with negative E-Cad expression (IHC, ×400); (b) patients with positive E-Cad expression (IHC, ×400); (c) patients with negative Ki-67 expression (IHC, ×400); (d) patients with positive Ki-67 expression (IHC, ×400); (e) patients with negative CK20 expression (IHC, ×400); (f) patients with positive CK20 expression (IHC, ×400). → (red) CD8^+^/PD-1^+^ TILs; → (black) CD8^+^/PD-1^−^ TILs. CD8^+^/PD1^+^ TILs showed a red cytomembrane and brown cytoplasm, and CD8^+^/PD1^−^ TILs showed a red cytomembrane. Patients with negative E-Cad had less CD8^+^/PD-1^−^ TILs than positive patients; patients with negative Ki-67 had less CD8^+^/PD-1^+^ and CD8^+^/PD-1^−^ TILs than positive patients; and patients with negative CK20 expression had less CD8^+^/PD-1^−^ TILs than positive patients.

The expression of CK7 was not associated with cell counts of any phenotypes of CD8^+^ TILs (Table 5). The E-Cad expression percentile had a significant association with CD8^+^ TIL counts; patients with a positive E-Cad expression had 18 CD8^+^ TILs/HPF, and patients with a negative E-Cad expression had 7 CD8^+^ TILs/HPF (*p* < 0.05, Table 5). Cell counts of CD8^+^/PD1^−^ TILs, not CD8^+^/PD1^+^ TILs, were significantly related with E-Cad expression percentile, and there were 14 CD8^+^/PD1^−^ TILs in BC patients with a positive E-Cad expression (Figure 2(a)) and 6 CD8^+^/PD1^−^ TILs in BC patients with a negative E-Cad expression (*p* < 0.05, Figure 2(b), Table 5). The correlation coefficient was 0.23 (*p* < 0.05) between the Ki-67 index and count of CD8^+^ TILs (Table 5). Regarding the PD-1 expression, the Ki-67 index was significantly related with both cell counts of CD8^+^/PD1^+^ TILs and CD8^+^/PD1^−^TILs (Table 5). The correlation coefficients were 0.19 and 0.21, respectively (*p* < 0.05, Table 5). Patients with a Ki-67 expression had more counts of CD8^+^/PD1^+^ TILs and CD8^+^/PD1^−^ TILs in the tumor microenvironment (Figures 2(c) and 2(d)). The CK20 expression percentile had a significant relationship with cell counts of CD8^+^ TILs; patients with a positive CK20 expression have 39 CD8^+^ TILs/HPF, higher than in patients with a negative expression (20/HPF, *p* < 0.05, Table 5). Though the CK20 expression percentile was not correlated with cell counts of CD8^+^/PD1^+^TILs, the correlation was significant with cell counts of CD8^+^/PD1^−^ TILs and the median cell counts were 33/HPF and 16/HPF for positive and negative CK20 expressions, respectively (*p* < 0.05, Figures 2(e) and 2(f), Table 5).

## 5. Discussion

BC development, prognosis, and treatment efficacy were associated with the tumor microenvironment. CD8^+^/PD-1^+^ and CD8^+^/PD-1^−^ TILs had associations with different clinic-pathological characteristics. Cell counts of CD8^+^/PD-1^−^ TILs had a significant relationship with younger age and negative axillary lymph node metastasis. CK20 and E-Cad expression was associated with a higher number of CD8^+^/PD-1^−^ TILs. The Ki-67 index was correlated with both CD8^+^/PD-1^+^ and CD8^+^/PD-1^−^ TILs. The BC immune microenvironment had a variety of TIL phenotypes, and each phenotype had particular biological functions and prognostic features.

PD-1 is a member of the CD28/CTLA-4 family of costimulatory receptors, transmits an inhibitory signal to the T cell, suppresses immune responses, and commits to the immune tolerance and functional exhaustion on T cells [8, 13]. Helper T cells, cytotoxic T cells, regulatory T cells, follicular T and B cells, and antigen-presenting cells all had a PD-1 expression on the cytomembrane [14]. CD8^+^ T cells from chronic lymphocytic leukemia patients had a significantly higher PD-1 expression than healthy controls [15]. In addition, the activated CD8^+^ T cells from chronic lymphocytic leukemia patients had a higher PD-1 expression significantly [15]. The number of CD8^+^ TILs was similar between pancreatic tumor tissues and adjacent nontumor tissues; however, the number of CD8^+^/PD-1^+^ TILs was significantly higher in tumor tissues [16]. Additionally, a high PD-1 expression on CD8^+^ TILs was related with node metastasis, distant metastasis, and clinical stage [16]. The exhausted CD8^+^ T lymphocytes produced low levels of IL-2, TNF-*α*, and IFN-*γ* and experienced a lower proliferation activity [17, 18]. The PD-1/PD-L1 crosstalk contributed to the low production of cytokines from CD8^+^ T cells, and blocking of the crosstalk by the anti-PD-L1 antibody increased the secretion [15]. The PD-1/PD-L1 signaling pathway maintained an immunosuppressive tumor microenvironment, which contributed to T cell dysfunction and attenuated antitumor immunity.

Ki-67 is a proliferative cell nuclear antigen, and the Ki-67 index correlates with the cell mitotic cycle. Ki-67 was expressed in all phases except G0 and early G1 and had the peak level in the M period [19]. The Ki-67 index was an indicator of malignant degree and proliferation activity and an independent prognostic factor for BC recurrence and survival [20]. PD-1 expression on effector T cells exhausted the antitumor immunity, modulated the cytokine secretion, and compromised the control of tumor growth [11]. PD-1 signaling affected the tumor chemoresistance and metastasis [21]. CD8^+^/CD103^+^/PD-1^+^ TILs were correlated with a high expression of Ki-67 in non-small-cell lung carcinoma [22]. In this study, CD8^+^/PD-1^+^ TILs were correlated with a high Ki-67 index. BC expressing high levels of Ki-67 had more exhausted CD8^+^ T cells in the tumor microenvironment and was related with a suppressive immune microenvironment.

A higher level of PD-1^+^ TIL was associated with poor prognosis in human BC [9]. A high expression level of PD-1 on CD8^+^ TILs was significantly correlated with poor survival of pancreatic ductal adenocarcinoma patients [16]. High CD8^+^/PD-1^+^ TILs were associated with a poor survival time for non-small-cell lung carcinoma; however, high CD8^+^/PD-1^−^ TILs were associated with a longer survival time [23]. In this study, we found that BC patients with high CD8^+^/PD-1^−^ TILs were more likely to be young and free of axillary lymph node metastasis. It indicated the dysfunctional effector cells of CD8^+^/PD-1^+^ TILs and functional effector cells of CD8^+^/PD-1^−^ TILs. The patients receiving nivolumab treatment had a reduction in CD8^+^/PD-1^+^ TILs, and lower CD8^+^/PD-1^+^ TILs correlated with a prolonged progression-free survival [23].

E-Cad was responsible for calcium-mediated cell-to-cell adhesion [24]. E-Cad promoted intercellular adhesion between homogeneous cells and inhibited the infiltration and metastasis of tumor cells [25]. Downregulation of E-Cad contributed to exacerbation of tumor grade and stage and promoted the transition from adenoma to carcinoma [26]. E-Cad was a marker of epithelial phenotype, and downregulation of E-Cad was a marker of epithelial-mesenchymal transition (EMT) [27]. In esophageal cancer cell lines, PD-1 binding to PD-L1 reduced E-Cad expression and enhanced the EMT process [28]. Mesenchymal and epithelial-mesenchymal phenotypes of lung adenocarcinoma had a higher number of PD-1^+^ TILs than epithelial phenotypes [29]. We found that BC patients with E-Cad expression had more CD8^+^/PD-1^−^ TILs and an active immune microenvironment.

A small sample size was one limitation of our study. Secondly, we did not analyze intratumoral and stromal TILs separately. Third, IHC was the sole detection method and flow cytometry was not performed to detect TIL phenotypes. The number of CD8^+^/PD1^+^ TILs was too small. Some variables including CK7, CK20, and axillary lymph node metastasis missed the value in analysis.

## 6. Conclusion

The Ki-67 index had a significant correlation with both cell counts of functional and dysfunctional CD8^+^ TILs. E-Cad and CK20 expression was correlated with cell counts of functional CD8^+^ TILs. Further studies were warranted to explore the causal relationship.

## Figures and Tables

**Table 1:** The characteristics of patients.

Items	
Age, mean ± SD (*n* = 133)	57.8 ± 13.6
Histological grade, *n* (%)	
I	14 (11.3)
II	82 (66.1)
III	28 (22.6)
Vascular invasion, *n* (%)	
No	86 (67.7)
Yes	41 (32.3)
Nerve invasion, *n* (%)	
No	101 (82.1)
Yes	22 (17.3)
Axillary lymph node metastasis, *n* (%)	
No	22 (45.8)
Yes	26 (54.2)
E-Cadherin expression, *n* (%)	
No	6 (5.1)
Yes	112 (94.9)
Ki-67 index, mean ± SD (*n* = 125)	30%±25%
CK20 expression, *n* (%)	
No	56 (91.8)
Yes	5 (8.2)
CK7 expression, *n* (%)	
No	9 (13.2)
Yes	59 (86.8)

**Table 2:** The relationship between the proportion of CD8/PD-1 TILs and clinical characteristics.

	Proportion of CD8^+^ TILs (%)	*p*	Proportion of CD8^+^/PD-1^+^ TILs (%)	*p*	Proportion of CD8^+^/PD1^−^TILs (%)	*p*
Age, median (IQR)^∗^		0.135		0.430		0.139
≤55	30 (10)		3 (5)		20 (13)	
>55	20 (20)		2 (6)		18 (15)	
Histological grade, median (IQR)^∗∗^		0.452		0.322		0.749
I	30 (32)		3 (10)		26 (22)	
II	20 (20)		2 (6)		18 (14)	
III	30 (20)		4 (6)		24 (17)	
Vascular invasion, median (IQR)^∗^		0.122		0.156		0.237
No	20 (20)		3 (5)		20 (17)	
Yes	20 (15)		2 (4)		18 (13)	
Nerve invasion, median (IQR)^∗^		0.706		0.982		0.706
No	20 (20)		3 (5)		18 (17)	
Yes	20 (10)		3 (5)		19 (11)	
Axillary lymph node metastasis, median (IQR)^∗^		*0.032*		0.335		*0.018*
No	30 (23)		4 (8)		24 (16)	
Yes	20 (20)		2 (5)		16 (13)	

^∗^Wilcoxon test. ^∗∗^Spearman correlation test.

**Table 3:** The relationship between cell counts of CD8/PD-1 TILs and clinical characteristics.

	CD8^+^ TILs	*p*	CD8^+^/PD1^+^TILs	*p*	CD8^+^/PD1^−^TILs	*p*
Age, median (IQR)^∗^		*0.01*		0.104		*0.008*
≤55	24 (27)		3 (7)		18 (22)	
>55	12 (18)		2 (4)		9 (14)	
Histological grade, median (IQR)^∗∗^		0.096		0.14		0.158
I	14 (19)		2 (7)		12 (13)	
II	18 (24)		2 (4)		14 (20)	
III	26 (24)		3 (13)		20 (23)	
Vascular invasion, median (IQR)^∗∗^		0.965		0.372		0.823
No	16 (26)		3 (6)		13 (21)	
Yes	18 (23)		2 (4)		15 (18)	
Nerve invasion, median (IQR)^∗^		0.459		0.628		0.567
No	18 (23)		22 (6)		14 (18)	
Yes	24 (27)		2 (10)		12 (25)	
Axillary lymph node metastasis, median (IQR)^∗^		0.051		0.159		*0.049*
No	33 (38)		4 (9)		27 (31)	
Yes	15 (23)		2 (5)		11 (16)	

^∗^Wilcoxon test. ^∗∗^Spearman correlation test.

**Table 4:** Correlation between proportion of CD8/PD-1 TILs and other molecules in breast cancer.

	Proportion of CD8^+^ TILs (%)	*p*	Proportion of CD8^+^/PD-1^+^ TILs (%)	*p*	Proportion of CD8^+^/PD1^−^TILs (%)	*p*
CK7, median (IQR)^∗^		0.940		0.549		0.971
No	30 (20)		3 (7)		21 (18)	
Yes	20 (10)		4 (7)		20 (14)	
CK20, median (IQR)^∗^		0.229		0.868		0.114
No	20 (10)		4 (7)		20 (13)	
Yes	30 (10)		4 (5)		24 (12)	
E-Cad, median (IQR)^∗^		0.271		0.606		0.262
No	15 (15)		2 (7)		12 (13)	
Yes	20 (10)		2 (5)		18 (17)	
Ki-67 index, correlation coefficient^†^	0.10	0.289	0.10	0.292	0.06	0.482

^∗^Wilcoxon test. ^†^Spearman correlation test.

**Table 5:** Correlation between cell counts of CD8/PD-1 TILs and other molecules in breast cancer.

	CD8^+^ TILs	*p*	CD8^+^/PD1^+^TIL s	*p*	CD8^+^/PD1^−^TILs	*p*
CK7, median (IQR)^∗^		0.993		0.745		0.856
No	27 (40)		4 (7)		19 (36)	
Yes	20 (30)		3 (7)		16 (24)	
CK20, median (IQR)^∗^		*0.042*		0.362		*0.025*
No	20 (27)		3 (7)		16 (24)	
Yes	39 (50)		8 (10)		33 (41)	
E-Cad, median (IQR)^∗^		*0.043*		0.211		*0.045*
No	7 (11)		2 (4)		6 (8)	
Yes	18 (24)		3 (6)		14 (21)	
Ki-67 index, correlation coefficient^†^	0.23	*0.009*	0.19	*0.036*	0.21	*0.022*

^∗^Wilcoxon test. ^†^Spearman correlation test.

## Data Availability

The data used to support the findings of this study are available from the corresponding author upon request.

## References

[1] Chen W., Sun K., Zheng R. (2018). Cancer incidence and mortality in China, 2014. *Chinese journal of cancer research*.

[2] Zheng S., Bai J. Q., Li J. (2012). The pathologic characteristics of breast cancer in China and its shift during 1999-2008: a national-wide multicenter cross-sectional image over 10 years. *International journal of cancer*.

[3] Garcia-Martinez E., Gil G. L., Benito A. C. (2014). Tumor-infiltrating immune cell profiles and their change after neoadjuvant chemotherapy predict response and prognosis of breast cancer. *Breast cancer research*.

[4] Gajewski T. F., Schreiber H., Fu Y. X. (2013). Innate and adaptive immune cells in the tumor microenvironment. *Nature Immunology*.

[5] Seo A. N., Lee H. J., Kim E. J. (2013). Tumour-infiltrating CD8+ lymphocytes as an independent predictive factor for pathological complete response to primary systemic therapy in breast cancer. *British journal of cancer*.

[6] Chamoto K., Al-Habsi M., Honjo T. (2017). Role of PD-1 in immunity and diseases. *Current Topics in Microbiology and Immunology*.

[7] Dong H., Strome S. E., Salomao D. R. (2002). Tumor-associated B7-H1 promotes T-cell apoptosis: a potential mechanism of immune evasion. *Nature medicine*.

[8] Flies D. B., Sandler B. J., Sznol M., Chen L. (2011). Blockade of the B7-H1/PD-1 pathway for cancer immunotherapy. *The Yale journal of biology and medicine*.

[9] Muenst S., Soysal S. D., Gao F., Obermann E. C., Oertli D., Gillanders W. E. (2013). The presence of programmed death 1 (PD-1)-positive tumor-infiltrating lymphocytes is associated with poor prognosis in human breast cancer. *Breast cancer research and treatment*.

[10] Ahmadzadeh M., Johnson L. A., Heemskerk B. (2009). Tumor antigen-specific CD8 T cells infiltrating the tumor express high levels of PD-1 and are functionally impaired. *Blood*.

[11] Liu X., Gibbons R. M., Harrington S. M. (2013). Endogenous tumor-reactive CD8^+^ T cells are differentiated effector cells expressing high levels of CD11a and PD-1 but are unable to control tumor growth. *Oncoimmunology*.

[12] Wang R., Shi F., Zhao L., Zhao Y., Wu G., Song Q. K. (2018). High expression of E-cadherin and Ki-67 associated with functional/dysfunctional phenotypes of tumor-infiltrating lymphocytes among Chinese patients with operable breast cancer. *The Journal of International Medical Research*.

[13] Bour-Jordan H., Esensten J. H., Martinez-Llordella M., Penaranda C., Stumpf M., Bluestone J. A. (2011). Intrinsic and extrinsic control of peripheral T-cell tolerance by costimulatory molecules of the CD28/ B7 family. *Immunological Reviews*.

[14] Yao S., Chen L. (2014). PD-1 as an immune modulatory receptor. *Cancer Journal*.

[15] Brusa D., Serra S., Coscia M. (2013). The PD-1/PD-L1 axis contributes to T-cell dysfunction in chronic lymphocytic leukemia. *Haematologica*.

[16] Shen T., Zhou L., Shen H. (2017). Prognostic value of programmed cell death protein 1 expression on CD8+ T lymphocytes in pancreatic cancer. *Scientific Reports*.

[17] Taghiloo S., Allahmoradi E., Tehrani M. (2017). Frequency and functional characterization of exhausted CD8+ T cells in chronic lymphocytic leukemia. *European journal of haematology*.

[18] Wang M., Bu J., Zhou M. (2018). CD8^+^ T cells expressing both PD-1 and TIGIT but not CD226 are dysfunctional in acute myeloid leukemia (AML) patients. *Clinical Immunology*.

[19] MacCallum D. E., Hall P. A. (2000). The location of pKi67 in the outer dense fibrillary compartment of the nucleolus points to a role in ribosome biogenesis during the cell division cycle. *The Journal of pathology*.

[20] Yerushalmi R., Woods R., Ravdin P. M., Hayes M. M., Gelmon K. A. (2010). Ki67 in breast cancer: prognostic and predictive potential. *The Lancet Oncology*.

[21] Black M., Barsoum I. B., Truesdell P. (2016). Activation of the PD-1/PD-L1 immune checkpoint confers tumor cell chemoresistance associated with increased metastasis. *Oncotarget*.

[22] Wang P., Huang B., Gao Y. (2018). CD103^+^CD8^+^ T lymphocytes in non-small cell lung cancer are phenotypically and functionally primed to respond to PD-1 blockade. *Cellular immunology*.

[23] Mazzaschi G., Madeddu D., Falco A. (2018). Low PD-1 expression in cytotoxic CD8^+^ tumor-infiltrating lymphocytes confers an immune-privileged tissue microenvironment in NSCLC with a prognostic and predictive value. *Clinical cancer research*.

[24] Liu Y., Zhao J., Zhang P. Y. (2012). MicroRNA-10b targets E-cadherin and modulates breast cancer metastasis. *Medical science monitor*.

[25] Adhikary A., Chakraborty S., Mazumdar M. (2014). Inhibition of epithelial to mesenchymal transition by E-cadherin up-regulation via repression of slug transcription and inhibition of E-cadherin degradation: dual role of scaffold/matrix attachment region-binding protein 1 (SMAR1) in breast cancer cells. *The Journal of biological chemistry*.

[26] Ye X., Weinberg R. A. (2015). Epithelial-mesenchymal plasticity: a central regulator of cancer progression. *Trends in cell biology*.

[27] Zheng G., Lyons J. G., Tan T. K. (2009). Disruption of E-cadherin by matrix metalloproteinase directly mediates epithelial-mesenchymal transition downstream of transforming growth factor-*β*1 in renal tubular epithelial cells. *The American journal of pathology*.

[28] Chen L., Xiong Y., Li J. (2017). PD-L1 expression promotes epithelial to mesenchymal transition in human esophageal cancer. *Cellular physiology and biochemistry*.

[29] Kim S., Koh J., Kim M. Y. (2016). PD-L1 expression is associated with epithelial-to-mesenchymal transition in adenocarcinoma of the lung. *Human pathology*.

